# Supplementary material for: Modeling glioblastoma heterogeneity as a dynamic network of cell states
Source: Mol Syst Biol. 2021 Sep 16;17(9):e10105. doi: 10.15252/msb.202010105 (PMC8444284; doi:10.15252/msb.202010105)
Supplement: Supplementary file 6 — Source Data for Figure 5 [file MSB-17-e10105-s004.zip › Figure5A_sourcedata/GSEA_3071/nosigdb_stateIII.GseaPreranked.1622795155562/gsea_report_for_na_pos_1622795155562.html]

Report for na\_pos 1622795155562 [GSEA]

| GS  follow link to MSigDB | GS DETAILS | SIZE | ES | NES | NOM p-val | FDR q-val | FWER p-val | RANK AT MAX | LEADING EDGE || 1 | NB\_KI\_CLUSTER1 | Details ... | 29 | 0.92 | 5.94 | 0.000 | 0.000 | 0.000 | 324 | tags=100%, list=8%, signal=108% |
| 2 | RICHARDS\_NOWAKOWSKI\_SCIENCE\_2017\_NIN2\_UPREG | Details ... | 169 | 0.36 | 5.69 | 0.000 | 0.000 | 0.000 | 399 | tags=49%, list=10%, signal=52% |
| 3 | RICHARDS\_NOWAKOWSKI\_SCIENCE\_2017\_MGE\_RG2\_UPREG | Details ... | 197 | 0.31 | 4.85 | 0.000 | 0.000 | 0.000 | 394 | tags=42%, list=10%, signal=44% |
| 4 | NB\_KI\_CLUSTER3 | Details ... | 34 | 0.56 | 4.13 | 0.000 | 0.000 | 0.000 | 316 | tags=65%, list=8%, signal=70% |
| 5 | DOROSZKO\_U3054MG\_CLUSTER\_9 | Details ... | 92 | 0.32 | 3.90 | 0.000 | 0.000 | 0.000 | 454 | tags=48%, list=12%, signal=53% |
| 6 | RICHARDS\_NOWAKOWSKI\_SCIENCE\_2017\_IPC\_NEN3\_UPREG | Details ... | 120 | 0.25 | 3.47 | 0.000 | 0.000 | 0.000 | 295 | tags=34%, list=8%, signal=36% |
| 7 | LARSSON\_STATE\_1 | Details ... | 48 | 0.39 | 3.38 | 0.000 | 0.000 | 0.000 | 384 | tags=60%, list=10%, signal=66% |
| 8 | LARSSON\_STATE\_5 | Details ... | 65 | 0.26 | 2.75 | 0.000 | 0.000 | 0.000 | 178 | tags=31%, list=5%, signal=32% |
| 9 | DOROSZKO\_U3180MG\_CLUSTER\_3 | Details ... | 122 | 0.18 | 2.51 | 0.000 | 0.001 | 0.005 | 513 | tags=35%, list=13%, signal=39% |
| 10 | NB\_KI\_CLUSTER11 | Details ... | 19 | 0.29 | 1.59 | 0.048 | 0.041 | 0.371 | 373 | tags=37%, list=9%, signal=41% |
| 11 | RICHARDS\_NOWAKOWSKI\_SCIENCE\_2017\_GLYC\_UPREG | Details ... | 237 | 0.10 | 1.39 | 0.000 | 0.100 | 0.719 | 524 | tags=27%, list=13%, signal=29% |
| 12 | NB\_KI\_CLUSTER9 | Details ... | 22 | 0.17 | 1.00 | 0.447 | 0.434 | 0.999 | 2052 | tags=77%, list=52%, signal=161% |
| 13 | FELDMAN\_2020\_LOWRNA | Details ... | 379 | 0.30 |  |  | 1.000 | 0.000 | 429 | tags=41%, list=11%, signal=42% |
| 14 | RICHARDS\_NOWAKOWSKI\_SCIENCE\_2017\_CHOROID\_UPREG | Details ... | 220 | 0.24 |  |  | 1.000 | 0.000 | 371 | tags=34%, list=9%, signal=35% |
| 15 | RICHARDS\_NOWAKOWSKI\_SCIENCE\_2017\_IPC\_DIV2\_UPREG | Details ... | 296 | 0.14 |  |  | 1.000 | 0.000 | 391 | tags=25%, list=10%, signal=25% |
| 16 | RICHARDS\_NOWAKOWSKI\_SCIENCE\_2017\_MGE\_DIV\_UPREG | Details ... | 208 | 0.17 |  |  | 1.000 | 0.000 | 402 | tags=29%, list=10%, signal=31% |
| 17 | RICHARDS\_NOWAKOWSKI\_SCIENCE\_2017\_RG\_EARLY\_UPREG | Details ... | 196 | 0.37 |  |  | 1.000 | 0.000 | 394 | tags=47%, list=10%, signal=50% |
| 18 | RICHARDS\_NOWAKOWSKI\_SCIENCE\_2017\_U3\_UPREG | Details ... | 249 | 0.21 |  |  | 1.000 | 0.000 | 445 | tags=33%, list=11%, signal=34% |
Table: Gene sets enriched in phenotype **na **[plain text format]****
